# On the incidence and prevalence of child maltreatment: a research agenda

**DOI:** 10.1186/s13034-016-0105-8

**Published:** 2016-06-14

**Authors:** Andreas Jud, Jörg M. Fegert, David Finkelhor

**Affiliations:** Department of Child and Adolescent Psychiatry/Psychotherapy, University of Ulm, Ulm, Germany; School of Social Work, Lucerne University of Applied Sciences and Arts, Lucerne, Switzerland; Crimes Against Children Research Center, University of New Hampshire, Durham, USA

## Abstract

Research on child maltreatment epidemiology has primarily been focused on population surveys with adult respondents. Far less attention has been paid to analyzing reported incidents of alleged child maltreatment and corresponding agency responses. This type of research is however indispensable to know how well a child protection system works and if the most vulnerable are identified and served. Notable findings of child maltreatment epidemiological research are summarized and directions for future studies discussed.

## Background^1^

Child maltreatment^2^ can have a devastating impact on children; adverse psychological, somatic and social consequences that affect childhood and later adult development and even persist into old age (e.g., [4–9]).

There is widespread agreement that in order to make progress in the prevention and reduction of child maltreatment it is important for policy-makers to have information on its scope and characteristics^3^. Researchers around the world have typically responded to this need using surveys to count the prevalence of child maltreatment in the general population. Hundreds of such studies have been done in dozens of countries and subordinate jurisdictions. They often associate the prevalence of victimization in childhood with (long-term) health and social outcomes in the adult population.

However, general population surveys have limited implications for specific policies in child protection. What policy-makers need most is information about which officials or agencies in their jurisdictions have knowledge of the problem, and what they are doing or not doing when they encounter it. Based on this information they can make concrete plans about how to allocate resources, change practices, train officials, and reorganize systems to better respond. They need information on whether these cases are coming to the attention of school teachers or police or doctors and what these professionals are doing. It may turn out that some officials are encountering very few cases; perhaps they need more training. It may turn out that other officials are finding cases but failing to do anything about them. Or cases that would be best dealt with by doctors are instead primarily coming to the attention of teachers but not getting referred. This knowledge can promote strategies for change. As policy-makers make changes, provide training, and raise awareness, they will then want to know if their reforms are changing the patterns they originally observed.

The most useful studies for policy-makers are the ones with information about the agencies and officials who are in positions to help and respond. In comparison to population surveys, where children and families are surveyed directly, “agency surveys” collect data from community and government organizations involved with children, such as schools, law enforcement, hospitals, mental health agencies, family service agencies, NGOs, and child protection agencies. This commentary will address need for future research for the relative wealth of population surveys and identify a framework for improving research on agency response to child maltreatment.

## Population surveys

Since Finkelhors' review of international epidemiology on child sexual abuse in 1994 [11], prevalence studies on child sexual abuse have been meta-analyzed repeatedly [12–14]. Stoltenborgh et al. [14] included 331 independent samples in their meta-analysis with a total of around 10 million participants. While prevalence rates on child sexual victimization varied notably around 12.7 % (95 % CI 10.7–15.0 %), a significantly higher rate of victimized females was widely but not universally observed. Findings on regional differences, socioeconomic development of a nation and on other indicators have been less clear-cut [12, 14]. A large part of the variation remains unexplained and differences are to some degree due to varying definitions and methodological artefacts. Small sample size, non-random design, low return rate and large number of items tend to increase the prevalence rate of a study [12, 14]. At the least representative samples should be a sine qua non for prevalence surveys.

In a recent series, Stoltenborgh and colleagues [15–17], have also reviewed surveys on the prevalence of neglect, physical maltreatment and emotional abuse. Like the findings on child sexual abuse, the variation in prevalence rates for other forms of child maltreatment is vast, too; definitional disparities and methodological artifacts are important contributors to variation. However, no skewed gender distribution is reported outside of child sexual abuse. A ‘neglect of neglect’ (e.g., [18]) is still evident in research on child maltreatment with the review on neglect being able to summarize only 16 studies [16].

Beside definitional issues that affect all research on child maltreatment and will be addressed below, two recommendations are offered for the relatively well trodden path of population surveys. Most population surveys are directed towards adult survivors of child maltreatment through telephone interviews or self-administered questionnaires (Stoltenborgh et al. [14–17]). Not only are the adult participants’ responses afflicted by memory biases, these retrospective studies also provide rates of maltreatment that apply to the past, often at least a decade ago. Self-reports of adolescents, on the other hand, provide a more current view on the scope of the problem and respondents’ memory is less affected by a long delay. In combination with studies on agency response to child maltreatment, only surveys with adolescents’ self-reports can provide accurate information on underserved populations. Furthermore, surveys with adolescents might provide a more accurate view on peer-to-peer violence (e.g., [19]). To address limitations of a particular source of information, researchers can also combine caregiver reports on child maltreatment with adolescents’ self-reports [20, 21]. Overall, the benefits of adolescent self-reports outweigh the added costs of preparing and managing a survey with legally minor participants. As a second recommendation, more attention should be given to including and/or oversampling high-risk populations [22, 23].

## Agency surveys and administrative data

While there is a solid body of research on measuring child maltreatment prevalence through self-report surveys, far less attention has been paid to studying incidents of child maltreatment known to agencies (cf. [24]). However, a few countries, such as the United States, New Zealand, and the Netherlands, have collected data on how their service agencies are responding to child maltreatment, mainly using two distinct data collection strategies: professional surveys and/or administrative data extraction (cf. [25]).

Globally, only three cross-sectional professional surveys are currently conducted on a cyclical basis on the nature and the extent of child maltreatment: the (U.S.) National Incidence Study of Child Abuse and Neglect (NIS) (e.g., [26, 27]), the Canadian Incidence Study of Reported Child Abuse and Neglect (CIS) (e.g., Public Health Agency of Canada [28, 29]), and the Netherlands nationwide prevalence study of child maltreatment (NPM) (e.g., [30]). All three surveys rely on data obtained from nationally representative samples of child protective services workers during a 3-month reference period. Representativeness is achieved through a universal inclusion strategy or stratified random sampling of child protective services. The incidents are extrapolated to an estimate of the annual national prevalence rate of child maltreatment (cf. [31]). Additionally, the NIS and NPM also include survey data from frontline professionals in other agencies that have frequent contact with children—for example, hospitals, day care centers, mental health agencies, and municipal police departments. In contrast to several population surveys that sometimes rely on small and non-random samples, professional surveys apply generally more rigorous methodological standards.

Examples of child maltreatment research using country-wide administrative data sets are particularly rare. In the US, a national database on children and families who come to the attention of state public child welfare agencies was permanently established in the early 1990s [32, 33]. Child protection agencies across the US systematically enter child maltreatment case data into online databases. US states then regularly submit these data to the National Child Abuse and Neglect Data System (NCANDS). Participation of individual US states in the NCANDS system is voluntary, but funding incentives for system development has motivated participation; the data system currently includes all 50 states [33]. Other nationally representative agency surveys and country-wide administrative data sets (e.g., for Australia, the Republic of Korea or the United Kingdom) are addressed in a separate overview [34].

Agency data and population surveys agree on the finding of higher rates for female than male victims of child sexual abuse and equal gender distribution for other types of maltreatment. In agency data, incidents of child sexual abuse are generally the least prevalent form of child maltreatment with percentages often around 3–9 % (e.g., [27, 30]). The understudied phenomenon of child neglect, on the other hand, is by far the most prevalent form in agency data. Findings from agency data are also in agreement with surveys insofar as children are often not only subjected to one type of maltreatment, but multiple types– at the same time or by being victimized at different times in different contexts [35, 36].

## Trends

The NCANDS provides the longest-running data set to analyze trends. Finkelhor et al. [37] have noted that rates of child sexual abuse (−64 %) and child physical maltreatment (−55 %) have both markedly dropped since the early 1990s. The promising trend in agency reported cases of child sexual abuse has been corroborated by a concurring decrease shown in several prevalence studies [38]. The evidence from population surveys shows trends similar to the agency data on declines for physical maltreatment [37]. However, hospital data show no decline in maltreatment-related injuries or fatalities [39]. For neglect, the most prevalent form of child maltreatment, there is some smaller decline in the period since 2006 in agency cases. There are similar data from New Zealand [40].

## Costs of child maltreatment

Only a few studies have tried to estimate costs for a nation or region [41–45]. They concur in establishing child maltreatment as a serious public health issue that comes with large costs for a society. Indirect costs exceed the direct costs—loss of productivity has been identified as the most important element [44]. Definitional inconsistencies and methodological variations of underlying population surveys have led to a considerable variation of prevalence estimates and, consequently, estimates of child maltreatment costs. Habetha et al. [44] estimated 2008 per capita costs for Germany between 134.82 Euro and 363.58 Euro corresponding to 0.44 % (lower bound) or 1.2 % (upper bound) of Germany’s GDP. The lower bound is close to the Australian estimate [45], while the upper bound is close to the Canadian estimate [41].

The relevant impact of child maltreatment on public health becomes even more important if intergenerational transmission is considered as an ongoing element (e.g., [46]): There is an increased risk for the offspring of child maltreatment victims to themselves experience similar adverse events (cf. [47]).

## Recommendations

Child maltreatment comes with great costs for society and the need for more research on agency responses to child maltreatment has been stressed throughout this commentary. Progress in this area of research is however dependent on a collaborative effort between researchers, administrators, frontline staff and policy-makers. Building trust between these stakeholders is key for arriving at an effective knowledge-generating partnership. Trust is developed and nurtured through positive experiences and consistent contact [48]. Two major barriers have to be addressed to advance research on agency response to child maltreatment:

- The first—and probably the most important—step in this reciprocal and collaborative effort is a process of developing shared definitions between research and practice, e.g. through establishing a minimum data set that identifies a common set of variables for the tracking of child maltreatment [49]. This comprises measures of severity and chronicity of abuse to match the risk factors with future outcomes (e.g., [50]). Developing shared definitions is not just essential for research on agency response to child maltreatment, but also for future population surveys. This is especially important for neglect and psychological abuse as these types are harder to define and less conceptually clear than physical or sexual abuse (e.g., [51–53]). Only shared definitions will allow for increased comparability of findings on prevalence and reported incidents to identify gaps in service provision.
- Second, in our experience, the major barrier and biggest threat to participation of agencies in surveys is work burden [48]. Frontline workers in child protection are continuously struggling to allocate scarce resources to the most urgent problems (e.g., [54]). Extra work for data collection will conflict with work time for clients or with the worker’s free time. Workers need to perceive that the study is useful and important, and it is therefore essential to create a questionnaire that covers important issues while being brief, user-friendly and written in concise and clear language [48]. Innovative approaches to extracting data from files might also be developed [55].

Only more professional surveys will increase the relevant knowledge to identify gaps in service provision, to improve preventive efforts and increase opportunities for early intervention ([3], p. 3). If an evidence-base is lacking, initiatives to improve services for maltreated children are likely to not correspond with needs and rely on distorting factors such as media coverage or political sensibilities (e.g., [24]). These circumstances might be ones that have contributed to the ‘neglect of neglect’. In sum, without knowledge about agency response to child maltreatment, we lack information about whether the costly investments in child welfare and protection are actually reaching the ones who need it the most (e.g., [56]). The UN Committee on the Rights of the Child [57] concludes that the right of the child to freedom from all forms of violence calls for “establishing a comprehensive and reliable national data collection system in order to ensure systematic monitoring and evaluation of systems (impact analyses), services, programmes and outcomes based on indicators aligned with universal standards, […]”.

## Footnotes

1 This commentary is partly based on a report for the German Independent Commissioner for questions related to child sexual abuse [1]. Part of the development of the report was an international expert meeting in December 2014 in Berlin with the participation of David Finkelhor, Carl-Göran Svedin and Nico Trocmé [2].

2 We use the term child maltreatment to refer to both acts or series of acts of commission or omission by a parent or other caregiver that results in harm, potential for harm, or threat of harm to a child [3]. Subtypes included are child neglect and sexual, physical and psychological abuse.

3 The following two paragraphs have been slightly adapted from Jud et al. [10].
